# Miniscalpel-Needle versus Steroid Injection for Plantar Fasciitis: A Randomized Controlled Trial with a 12-Month Follow-Up

**DOI:** 10.1155/2014/164714

**Published:** 2014-07-08

**Authors:** Shuming Li, Tong Shen, Yongshan Liang, Ying Zhang, Bo Bai

**Affiliations:** ^1^Department of Rehabilitation Medicine, The First Affiliated Hospital of Guangzhou Medical University, No. 151, Yanjiang West Road, Guangzhou, Guangdong 510120, China; ^2^Department of Orthopaedics Medicine, The First Affiliated Hospital of Guangzhou Medical University, No. 151, Yanjiang West Road, Guangzhou, Guangdong 510120, China

## Abstract

Plantar fasciitis is the most common cause of heel pain in adults. A novel alternative medical instrument, the miniscalpel-needle (MSN), which is based on an acupuncture needle, has been recently developed in China. The objective of this study was to evaluate the effectiveness of the MSN release treatment versus that of traditional steroid injection for plantar fasciitis. Patients with plantar fasciitis were randomly assigned to 2 groups and followed up for 12 months, with 29 receiving MSN treatment and 25 receiving steroid injection treatment. The results showed that visual analog scale scores for morning pain, active pain, and overall heel pain all were decreased significantly in the MSN group from 1 to 12 months after treatment. In contrast, treatment with steroid injection showed a significant effect only at the 1-month follow-up but not at 6 or 12 months after treatment. Moreover, the MSN group achieved more rapid and sustained improvements than the steroid group throughout the duration of this study. No severe side effects were observed with MSN treatment. Our data suggest that the MSN release treatment is safe and has a significant benefit for plantar fasciitis compared to steroid injection.

## 1. Introduction

Plantar fasciitis commonly causes heel pain and affects approximately 10% of the general population [1]. Plantar fasciitis is characterized by pain and tenderness centered on the medial tubercle of the calcaneum upon weight bearing, especially immediately after a rest such as when getting out of bed in the morning [2]. Plantar fasciitis has been described as a self-limiting condition that will eventually resolve regardless of treatment [3, 4]. However, the condition can cause significant pain and disability for months or even years [5–7].

Current conventional treatments for plantar fasciitis include rest, nonsteroidal anti-inflammatory drugs (NSAIDs), physical therapy, stretch exercise, and steroid injection. Steroid injection is one of the most popular options used to treat this condition [8, 9]; however, it may produce serious side effects such as a recognized risk of subsequent plantar fascia rupture that has been reported by multiple studies [10–12]. Therefore, the exploration of alternative treatments is warranted.

**Figure 1:**
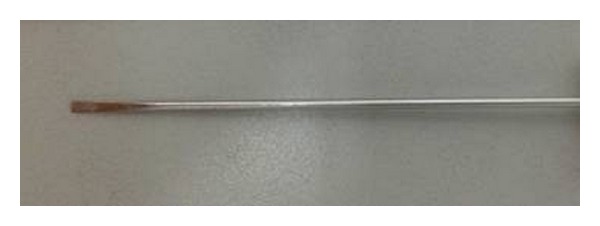
Photograph of the miniscalpel-needle.

Recently, new alternative medical instruments based on an acupuncture needle have been developed in China [13–17]. They are shaped like acupuncture needles and could be mainly classified as either a needle with a flat edge on the tip or a needle without a flat edge on the tip. Among these instruments, the miniscalpel-needle (MSN) is being increasingly used for many musculoskeletal pain conditions [13, 16]. The MSN is shaped like an acupuncture needle with a flat edge on the tip (Figure 1). Until now, only one clinical report investigated the use of MSN release for heel pain [18], but the study used no control group. Therefore, it is necessary to further evaluate the effectiveness of the MSN release treatment for plantar fasciitis. To the best of our knowledge, this is the first randomized, controlled study to compare the MSN release treatment with steroid injection for plantar fasciitis.

## 2. Materials and Methods

### 2.1. Participants

This randomized, controlled trial was approved by the ethical committee of the First Affiliated Hospital of the Guangzhou Medical University (trial registration: 2010-40). Patients were recruited in the outpatient clinic of the First Affiliated Hospital of the Guangzhou Medical University from July 2010 to July 2011. All patients were diagnosed based on X-ray imaging results, and 43 out of 61 patients had calcaneal spurs.

The allocation sequence was computer-generated with a simple randomization. The sequence was placed into sealed, consecutively numbered, and opaque envelops. Explanation of the trial was given to each patient. After informed consent was obtained, 61 patients were randomly allocated into the MSN group (*n* = 31) and the steroid injection group (*n* = 30). Two patients in the MSN group and 5 patients in the steroid injection group dropped out during the 12-month follow-up. In total, 54 patients completed the treatment protocols, and the 12-month follow-up included 29 patients in the MSN group and 25 patients in the steroid group. In the current study, all participants continued with their prior conservative treatment including physical therapy, stretching exercise, heel cushion, and NSAIDs if necessary.

### 2.2. Inclusion Criteria

Patients who were 18 to 70 years old and who had plantar fasciitis that failed to respond to at least 6 months of conservative treatments including physical therapy, NSAIDs, stretch exercise, and heel cushion were recruited. Patients were diagnosed as having plantar fasciitis if the heel pain was localized to the medial tubercle of the calcaneum, which is the site of the insertion of the plantar fascia and intrinsic muscles of the foot [2].

### 2.3. Exclusion Criteria

Patients were excluded if they had fracture or arthritis of the ankle and knee, previous foot surgery or trauma, nerve injury, a severe systemic disease, contralateral heel pain, or a history of MSN release treatment or local steroid injection into the heel pad or if they were pregnant.

### 2.4. Intervention

**Figure 2:**
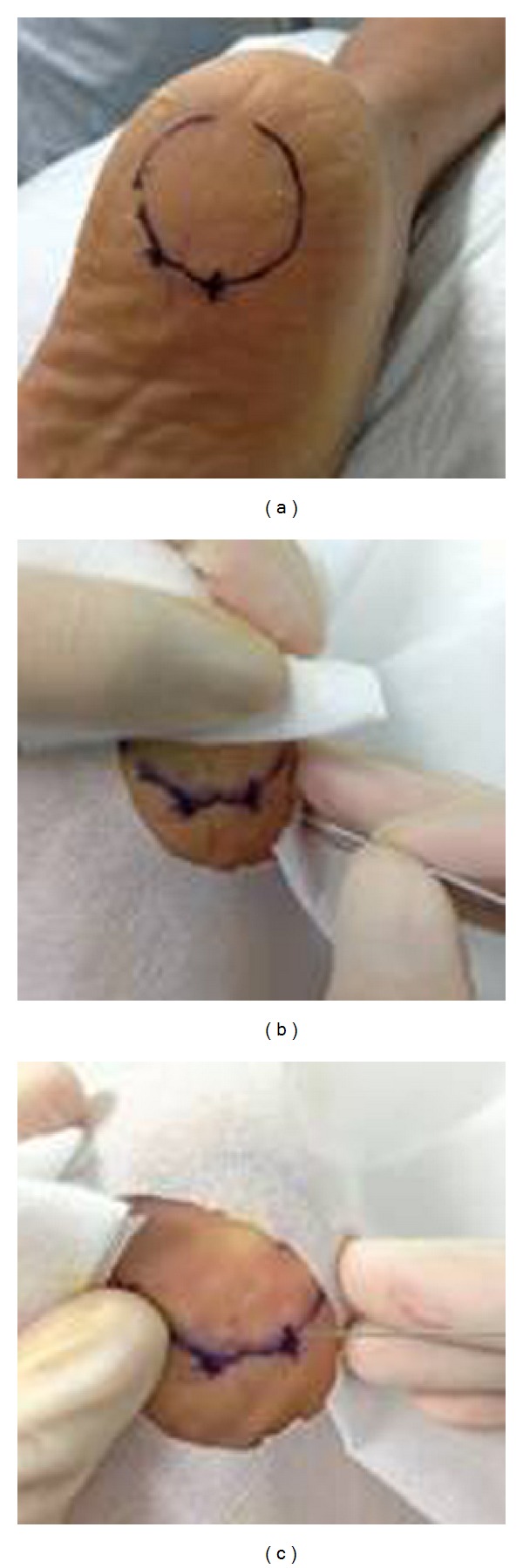
The MSN release treatment. (a) The use of a surface landmark at the most painful tender point for MSN release; (b) MSN release for plantar fasciitis; and (c) the initial wound post-MSN release.

The protocol for the MSN release treatment was based on that described in previous clinical reports [13–15]. The patient lay in a prone position with their feet hanging over the edge of a couch. The most painful tender point over the medial tubercle of the calcaneum was located and marked by palpating the heel (Figure 2(a)). After sterilization, the skin and subcutaneous tissues were anesthetized with 2 mL of 2% lidocaine. Then, the MSN (Figure 1, diameter 0.80 mm, length 50 mm, Huaxia Acupotomology Medical Equipment Factory, Beijing, China) was inserted into the tender point vertically with the direction of the MSN parallel to the long axis of the foot. The release of plantar fasciitis was performed by moving the MSN up and down 3–5 times without rotation (Figure 2(b)). Then, the MSN was withdrawn, and pressure was applied to the wound for 2 min to avoid bleeding (Figure 2(c)). The hole was covered with a simple adhesive bandage for 2 days.

The procedure of steroid injection was similar to that for MSN release. Briefly, 2 mL of 2% lidocaine plus 2 mL triamcinolone acetonide (20 mg) was injected into the most painful tender point. After treatment, the patients in both groups were observed for 30 min to record any adverse reaction. All patients were asked to avoid bearing weight on the heel pad for 2 days and examined at 1 month after treatment. The data from the 6- and 12-month follow-up period were collected by telephone interview.

### 2.5. Outcome Measures

The primary outcome measure was morning pain (the pain experienced during the first steps in the morning), which is a distinct feature of plantar fasciitis. Active pain (heel pain during activity) and the overall perception of heel pain were secondary outcomes. The pain was measured using the visual analog scale (VAS) between 0 and 10 points, in which 0 represented no pain and 10 represented the worst pain experienced by the patients [19, 20].

### 2.6. Statistical Analysis

All values presented are mean values ± standard deviation (SD). Baseline data of the 2 groups were compared by independent-sample *t*-test. Comparative analysis of categorical variables was performed using the Chi-square test. We used one-way ANOVA to analyze intergroup variability of the VAS scores. The analysis procedure was performed using IBM SPSS Statistics 19 software. Statistical significance was assumed if *P* < 0.05. Intention-to-treat analysis was performed with missing data being replaced by the last value carried forward.

## 3. Results

Plantar fasciitis patients were randomly assigned to 2 groups. In total, 54 patients completed one of the treatment protocols and the 12-month follow-up, with 29 patients in the MSN group and 25 patients in the steroid group. The baseline characteristics (age, sex, duration of symptoms, and VAS scores) are listed in Table 1. There were no significant differences in the baseline data between the two groups.

**Figure 3:**
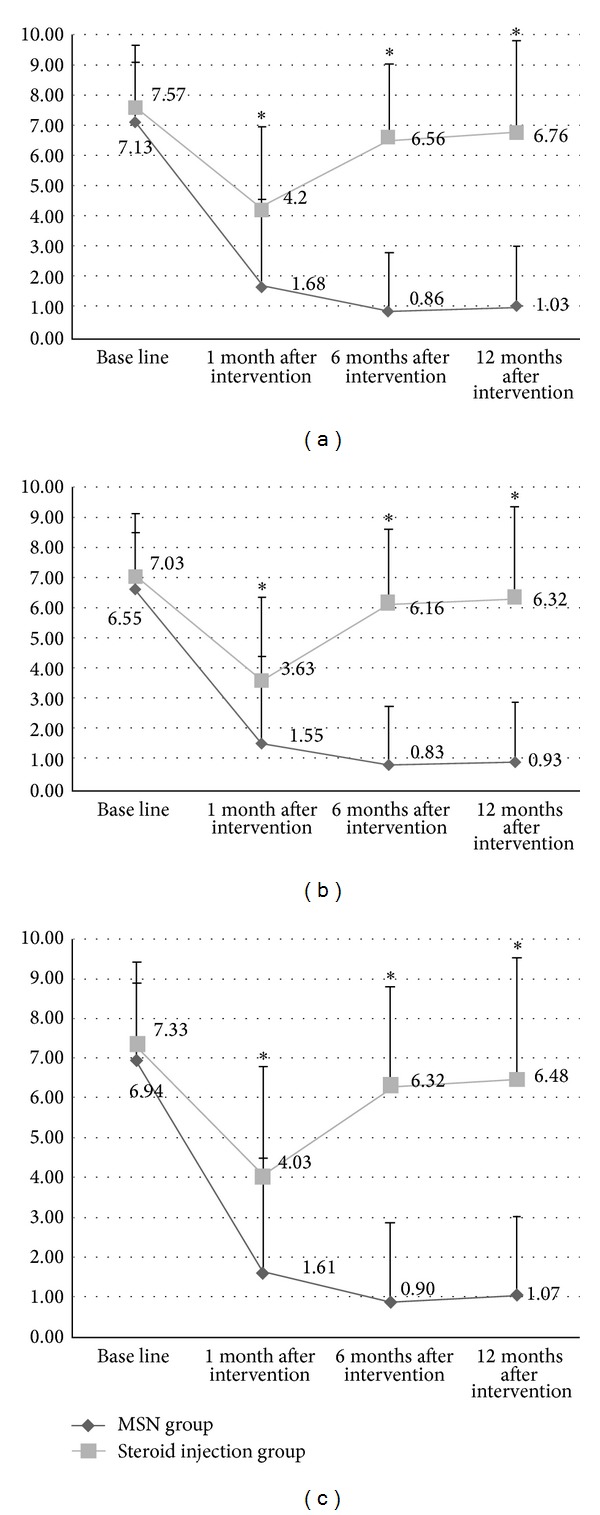
The effectiveness of MSN release treatment versus steroid injection for treating plantar fasciitis. (a) VAS scores for morning pain of MSN group decreased significantly compared to those of steroid injection group at 1-, 6-, and 12-month follow-up. (b) VAS scores for active pain of MSN group decreased significantly compared to those of steroid injection group at 1-, 6-, and 12-month follow-up. (c) VAS scores for overall pain of MSN group decreased significantly compared to those of steroid injection group at 1-, 6-, and 12-month follow-up. VAS: visual analog scale; MSN: miniscalpel-needle. *P < 0.05.

In the MSN group, the VAS scores for morning pain, active pain, and overall pain were significantly improved at 1, 6, and 12 months after intervention compared to the baseline scores (*P* < 0.01), but there were no statistical differences in the VAS scores observed between 1, 6, and 12 months after intervention (*P* > 0.05; Figure 3(a) and Table 2(a), Figure 3(b) and Table 2(b), and Figure 3(c) and Table 2(c)). In the steroid injection groups, the VAS scores for morning pain, active pain, and overall pain were significantly decreased only at 1 month after intervention (*P* < 0.01), but no significant improvement in pain was experienced at 6 or 12 months after intervention compared to the baseline levels (*P* > 0.05; Figure 3(a) and Table 2(a), Figure 3(b) and Table 2(b), and Figure 3(c) and Table 2(c)). Compared to steroid injection, the MSN treatment resulted in a statistically significant improvement in the VAS scores over the duration of the study (*P* < 0.01; Figure 3(a) and Table 2(a), Figure 3(b) and Table 2(b), and Figure 3(c) and Table 2(c)).

The side effects of the MSN release treatment were slight and included mild distending pain and subcutaneous bleeding at the treatment site. In total, 5 patients reported mild distending pain after intervention, and one patient experienced subcutaneous bleeding. However, the duration of all side effects was brief, and all patients recovered fully within 2 days.

## 4. Discussion

This is the first randomized, controlled study to evaluate and compare the effectiveness of the MSN release treatment and steroid injection for plantar fasciitis with a 12-month follow-up. The MSN group showed significantly reduced VAS scores for morning pain, active pain, and overall pain compared to the steroid injection group. Furthermore, the improvements of pain relief were maintained throughout the 12-month follow-up, suggesting that the MSN release treatment is superior to steroid injection for the long-term treatment of plantar fasciitis. In contrast, the steroid injection group showed statistically reduced VAS scores for morning pain, active pain, and overall pain only at 1 month after treatment, but not at 6 or 12 months, suggesting this treatment offers only short-term effectiveness.

In most cases, plantar fasciitis is described as a self-limiting condition that will eventually resolve regardless of treatment [3, 4]. In the current study, to avoid the effect of time on healing, the patients with chronic recalcitrant plantar fasciitis lasting 6 months or longer were recruited, with an average 8.81 ± 2.79 months in the MSN group and 9.80 ± 2.94 months in the steroid injection group. Furthermore, the patients failed to conservative treatments which included physical therapy modalities, NSAIDs, and heel cushion prior to enrollment. In addition, there were several studies showing that steroid injection was superior to placebo with short-term benefit [8, 9]. To support our findings in this study, a placebo controlled trial should be performed in the future study.

In current study, the use of prior conservative treatments was allowed. However, we recruited the patients with chronic recalcitrant plantar fasciitis failed to respond to the conservative treatments. So, these conservative treatments might not be a confounder in current study. In addition, 2 patients in the MSN group and 5 patients in the steroid infection group dropped out because of persistent heel pain in the period of follow-up.

It was reported that progressive plantar fasciitis and intrinsic foot muscle stretching techniques have been shown to reduce plantar fasciitis pain. Patients can be educated on how to perform foot and ankle stretches during physician office visit [4, 21]. Recently, another study reported that a combination of botulinum toxin A and plantar fascia stretching exercises yielded better results than intralesional steroids for patients with plantar fasciitis [22]. However, in order to assess the effectiveness of the MSN treatment and simplify the study, we focus on comparing the MSN treatment with the steroid infection for plantar fasciitis in current study. It would be worthwhile to compare the combination of the MSN with plantar fascia stretching exercises to the steroid injection for plantar fasciitis in future study.

The significance of calcaneal spurs in patients with plantar fasciitis has been questioned in some studies [23–25]. We found that 43 out of 61 patients had calcaneal spurs according to X-ray imaging results, which is in contrast to the results of a recent study [26]. However, it has also been reported that calcaneal spurs are of little diagnostic value due to the high prevalence of calcaneal spurs in asymptomatic patients [23, 27].

The MSN is one new alternative medicinal instrument that has been used to treat various disorders including cervical myofascial pain syndrome and trigger thumb [13–15]. The MSN release treatment has been used for performing minimal soft tissue dissection [16]. In this study, all patients had clear tender points at the plantar fasciitis. Most of the patients have no special feeling when the MSN was inserted into the tender point because the skin and subcutaneous tissues were anesthetized before the MSN treatment. Only some patients may feel distention at a tolerable level. The operator of MSN would feel heaviness or sense of resistance when the needle is inserted into adhesive tissue. The experience and technique of MSN are the key points to achieve the treatment effectiveness. The MSN release is both an acupuncture treatment and a microinvasive operation. Acupuncture is one of the most popular alternative and complementary medical treatments. Its success in pain syndromes has been demonstrated in many studies. The pain-relieving effects of acupuncture include central opioid pain inhibition [28], improvement of local circulation in a specific area [29–35], elimination of muscular spasm and tension [36, 37], and anti-inflammation [38–40]. On the other hand, MSN release is a microinvasive operation. The release may cut and detach the stiff and contractured plantar fasciitis, decrease the high tension of plantar fasciitis, and thus relieve the pain [18, 41]. For this reason, we believe that the MSN release treatment may be an effective treatment option for plantar fasciitis.

Steroid injection has been practiced widely, and some studies have shown good results with steroid injection for plantar fasciitis [8, 9, 42, 43]. In our steroid injection group, although significant improvements in VAS scores were observed 1 month after treatment, no statistical differences in VAS scores were detected at 6 or 12 months after intervention compared to baseline VAS scores. This limited, short-term benefit of steroid injection for plantar fasciitis might be due to many factors such as the baseline clinical characteristics of the patients included in this study. Similarly, Crawford et al. reported that steroid injection was superior to placebo at 1 month but not at subsequent follow-up assessments in their randomized controlled study involving 106 patients [8]. In another randomized study, Ball et al. examined 65 patients with plantar fasciitis and reported that steroid injection had significant short- and medium-term benefits [9].

Operative treatment provides a favorable effect in many studies [7, 44]. In a multisurgeon prospective analysis of 652 patients treated with endoscopic plantar fasciotomy, 97% of patients reported pain relief [45]. However, in addition to the long duration of postoperative recovery, some patients are at risk of serious side effects including reflex sympathetic dystrophy, flat foot or posterior tibial nerve injury, and calcaneocuboid and midtarsal joint pain [7, 46]. In this study, the side effects of the MSN release treatment included mild distending pain and subcutaneous bleeding at the treatment site. However, all side effects lasted for less than 2 days, and no severe side effects were reported during the 12-month follow-up. Because the MSN release treatment can be performed for minimal abnormal soft tissue dissension including plantar fasciitis, it has fewer side effects compared to the traditional surgery.

However, there were several limitations in this study. First, a drawback of this study is the lack of a true control group which may risk the confidence of the results. Second, the study could not keep patients blinded to the treatment type due to the nature of the interventions. Third, only subjective outcome measures were used to evaluate the effectiveness of the MSN release treatment for plantar fasciitis. Further study with a placebo controlled trial should be performed to assess the long-term effectiveness of the combination MSN with stretch exercise in the future.

## 5. Conclusion

In this study, patients who received MSN release treatment reported more favorable and more sustained improvements in pain compared to those who received steroid injection at 1-, 6- and 12-month follow-ups. Our data suggest that the MSN release treatment offers the advantages of effectiveness, convenience, and safety for patients who have failed to respond to conventional treatments for plantar fasciitis.

## Figures and Tables

**Table 1:** Baseline patient characteristics.

	MSN group (*n* = 31)	Steroid group (*n* = 30)	*P*
Sex (M/F)	10/19	7/25	0.27
Age (years)	54.74 ± 10.16	56.93 ± 9.25	0.38
Duration of symptoms (months)	8.81 ± 2.79	9.80 ± 2.94	0.18
VAS, morning pain	7.13 ± 1.82	7.57 ± 2.10	0.39
VAS, active pain	6.55 ± 1.75	7.03 ± 1.71	0.28
VAS, overall pain	6.94 ± 1.77	7.33 ± 2.09	0.43

**(a):** 

Morning pain	MSN group	Steroid injection group	*P* value
Baseline	7.13 ± 1.82	7.57 ± 2.10	0.387
1-month follow-up	1.68 ± 2.10	4.20 ± 2.47	0.000
6-month follow-up	0.86 ± 1.30	6.56 ± 2.40	0.000
12-month follow-up	1.03 ± 1.40	6.76 ± 2.70	0.000

**(b):** 

Active pain	MSN group	Steroid injection group	*P* value
Baseline	6.55 ± 1.75	7.03 ± 1.71	0.278
1-month follow-up	1.55 ± 1.95	3.63 ± 2.40	0.000
6-month follow-up	0.83 ± 1.63	6.16 ± 2.54	0.000
12-month follow-up	0.93 ± 1.70	6.32 ± 2.67	0.000

**(c):** 

Overall pain	MSN group	Steroid injection group	*P* value
Baseline	6.94 ± 1.77	7.33 ± 2.09	0.425
1-month follow-up	1.61 ± 2.14	4.03 ± 2.37	0.000
6-month follow-up	0.90 ± 1.72	6.32 ± 2.64	0.000
12-month follow-up	1.07 ± 1.69	6.48 ± 2.70	0.000
